# Towards Sustainable Wastewater Treatment: Bioindication as a Technique for Supporting Treatment Efficiency Assessment

**DOI:** 10.3390/ijerph191911859

**Published:** 2022-09-20

**Authors:** Justyna Drzymała, Joanna Kalka, Adam Sochacki, Ewa Felis

**Affiliations:** 1 The Biotechnology Centre, Silesian University of Technology, 44-100 Gliwice, Poland; 2 Environmental Biotechnology Department, Faculty of Energy and Environmental Engineering, Silesian University of Technology, Akademicka str 2A, 44-100 Gliwice, Poland; 3 Department of Applied Ecology, Faculty of Environmental Sciences, Czech University of Life Sciences Prague, 16500 Prague, Czech Republic

**Keywords:** environmental depollution, removal of emerging contaminants, bioindication, constructed wetlands, ecotoxicity, micropollutants, pharmaceutical pollution

## Abstract

Constructed wetlands (CWs) are a promising alternative for conventional methods of wastewater treatment. However, the biggest challenge in wastewater treatment is the improvement of the technology used so that it is possible to remove micropollutants without additional costs. The impact of wastewater treatment in CWs on toxicity towards *Aliivibrio fischeri*, *Daphnia magna* and *Lemna minor* was investigated. The effects of feeding regime (wastewater fed in five batches per week at a batch volume of 1 L, or twice per week at a batch volume of 2.5 L) and the presence of pharmaceuticals (diclofenac and sulfamethoxazole), as well as the presence of *Miscantus giganteus* plants in CW columns (twelve of the 24 columns that were planted) were analyzed. A reduction in toxicity was observed in all experimental setups. The effluents from constructed wetlands were classified as moderately toxic (average TU for *A. fischeri*, *D. magna* and *L. minor* was 0.9, 2.5 and 5.5, respectively). The feeding regime of 5 days of feeding/2 days of resting resulted in a positive impact on the ecotoxicological and chemical parameters of wastewater (removal of TOC, N-NH_4_ and pharmaceuticals). Extended exposure of *Miscantus giganteus* to the wastewater containing pharmaceuticals resulted in elevated activity of antioxidant enzymes (catalase and superoxide dismutase) in leaf material.

## 1. Introduction

Wastewater treatment plants based on conventional activated sludge technology or sequencing batch reactors and membrane bioreactors are not cost-efficient, energy balanced or sufficiently effective enterprises to remove micropollutants [1,2]. Constructed wetlands (CWs) are a promising alternative for conventional methods of wastewater treatment. CWs are artificial habitats made up of highly heterogeneous microbial communities and plants [3,4]. They provide significant water quality benefits and have a positive impact on wildlife habitat. CWs preserve and boost local biodiversity and can drive sustainable development [5,6,7]. The number of hydrophyte treatment plants increased significantly in the 1990s, and their use expanded to include the treatment of various types of wastewater (e.g., industrial). CWs have been used to restore habitat for native and migratory wildlife species, dispose of anthropogenic wastewater, control stormwater runoff, remediate brownfields and mitigate ecological disturbances resulting from the loss of naturally occurring wetlands. In addition, artificial wetlands have gained attention because of their economic and ecological benefits. Compared to conventional treatment technologies, CWs have proven to be an attractive and sustainable alternative due to their low costs and energy savings. Additionally, they provide natural habitats in urban and suburban areas, enhance aesthetic value in the local natural environment and are a favorable solution for small and medium-sized cities due to their easy operation and maintenance. Artificial wetlands are now widely used in many European countries [8,9].

Constructed wetland is a low-cost treatment technology; the operational and maintenance cost in constructed wetland is 1–2% of capital cost as compared to other technologies. Table 1 presents selected operational and maintenance costs attributed to CWs in comparison to SBR technology.

In general, constructed wetlands can be used to treat various types of wastewater with slightly lower removal efficiency and higher space requirements compared to other wastewater treatment technologies. Considering the low environmental impact of this type of facility and the elimination of the use of chemicals in the treatment process, as well as the lack of continuous supervision by the operator, this technology is definitely considered sustainable. However, the biggest challenge in wastewater treatment is the improvement of the technology used so that it is possible to remove micropollutants without incurring high additional costs. Micropollutants, such as pharmaceuticals, in household wastewater treatment are very difficult to remove and may exhibit a negative environmental impact [11]. The presence of micropollutants in the natural environment is largely due to aquatic reservoirs receiving treated wastewater [12,13]. Two of the most frequently detected pharmaceutical compounds in wastewater, surface water and groundwater are diclofenac (DCF) and sulfamethoxazole (SMX) [14]. DCF is a commonly used as a nonsteroidal anti-inflammatory drug (NSAID). Painkillers and anti-inflammatory drugs are the most commonly detected pharmaceuticals and personal care products (PPCPs) in wastewater due to their high consumption and over-the-counter availability [15]. In the European Union, DCF was included in the first watch list (WL) under the Water Framework Directive (WFD) [16,17]. The goal of the WL is to collect high-quality Union-wide monitoring data on potential water pollutants for the purpose of determining the risk they pose, and thus whether environmental quality standards (EQS) should be set for them at the EU level [18]. SMX is an antimicrobial sulfonamide used to suppress a wide spectrum of bacterial infections in human and veterinary medical practice [19]. The presence of SMX in the natural environment may be connected with bacterial resistance to antibiotics [20,21]. Moreover, SMX is one of the most often-detected xenobiotics in European wastewater treatment plants’ (WWTPs) effluents. According to the Felis et al. [20] the detection frequency for SMX in the effluents of 90 European WWTPs was 83%. The environmental risks resulting from the presence of pharmaceutical substances in wastewater emphasize a need for stringent monitoring of treated wastewater quality. However, current analytical procedures are complex, multistage and require sensitive measuring equipment. In practice, it means that the detection and determination of selected impurities, and in particular their transformation products (which may have higher toxicity than the parent compound), is not always possible. A more accessible and sensitive approach is needed for the monitoring of wastewater released to surface waters, and one that can monitor more than the standard physicochemical parameters.

Various strategies are used to regulate pharmaceutical residues in the environment [22]. The main technique is to monitor the concentrations of micropollutants in surface water, groundwater or treated sewage; however, as it was said before, these methods are often unable to detect all of the degradation products and are excessively expensive [23]. Another promising strategy might be bioindication—the use of aquatic organisms to monitor the safety level of sewage [24]. It seems reasonable to assume that wastewater, which does not pose a risk to natural organisms, can be safely discharged into the environment. Therefore, it is worth focusing not only on the detection of micropollutants in the environment but also on the assessment of the environmental risk caused by these substances.

Bioindication can be used to assess the ecotoxicological properties of wastewater and the effectiveness of the treatment process. The recommended procedure for the evaluation of environmental samples is to perform ecotoxicity tests with at least three bioindicators. Several studies have described the use of three model species representing different classes of bioindicator organisms: *Lemna minor*, representing “producers”; *Daphnia magna*, representing “consumers”; and *Aliivibrio fischeri*, representing “decomposers”. The *L. minor* plant is a macrophyte that is considered a useful bioindicator for ecotoxicological studies due to its small size, fast growth rate and sensitivity to numerous contaminants. The most favorable habitat for *L. minor* is water rich with decomposing organic matter, ensuring a constant supply of nutrients and trace elements [25,26]. The freshwater crustacean *D. magna* inhabits a variety of aquatic environments globally [27] and is widely used to monitor environmental pollution. Crustaceans are very important organisms from an ecological perspective due to their propensity for cleaning water reservoirs from algae, bacteria and protozoa. The luminescent, Gram-negative marine bacterium *A. fischeri* is commonly used in ecotoxicological studies [28]. The amount of light produced by *A. fischeri* is proportional to its metabolic activity, and any decrease in enzymatic activity results in inhibition of bioluminescence [29].

The aim of this investigation was to verify if the selected bioindication methods would be sustainable to determine the environmental risk caused by the presence of pharmaceuticals (DCF and SMX) in wastewater. For this purpose, the effect of raw and treated wastewater on three aquatic model organisms: *L. minor*, *D. magna* and *A. fischeri* were evaluated.

## 2. Materials and Methods

### 2.1. Tested Pharmaceuticals: Diclofenac and Sulfamethoxazole

DCF and SMX were purchased from Sigma-Aldrich (St. Louis, MA, USA) (purity > 99%). The properties of the tested pharmaceuticals as well as their environmental concentrations are listed in Appendix A—Table A1.

### 2.2. Experimental CW System

**Figure 1:**
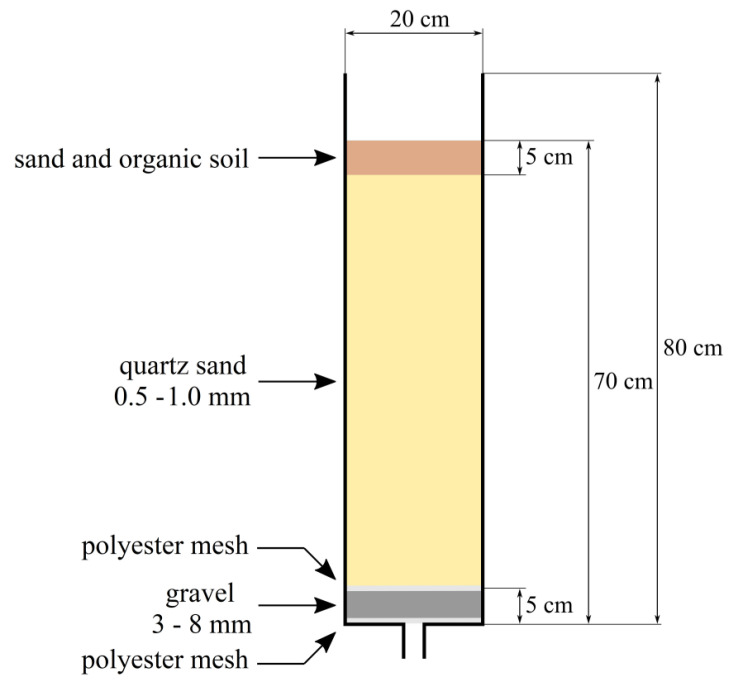
A scheme of experimental CW column.

**Figure 2:**
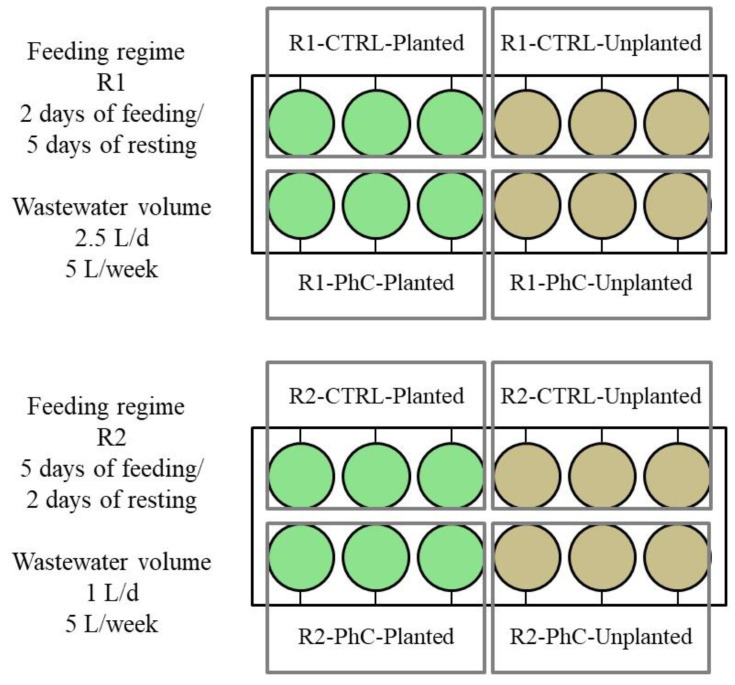
A scheme of the experimental system.

The laboratory system of vertical flow CWs consisted of 24 columns (each with a diameter of 0.2 m and height of 0.8 m, Figure 1 and Figure 2). In order to verify the effect of plants on the efficiency of wastewater treatment, including the removal of pharmaceuticals, half of the CW columns were planted with *Miscantus giganteus* marsh plants. The effect of the wastewater feeding regime, the presence of pharmaceuticals DCF and SMX (in concentrations 2 mg L^−1^ each) and the presence of *M. giganteus* on wastewater treatment processes, as well as the impact of these factors on ecotoxicity parameters, were investigated. The CW columns were set up in a heated room where the temperature was kept constant between 22–25 °C. The temperature was not adjusted during the study.

The studies presented in this work are a continuation of the preliminary research published by Sochacki et al. [30]. The results obtained in the preliminary studies were promising; therefore, it was decided to increase the concentration of pharmaceutical substances (from 0.5 to 2 mg L^−1^). Higher concentrations of the analyzed pharmaceuticals may occur in point sources of contamination, which undoubtedly are wastewater from single houses or from hospitals [15,20]. Since environmental micropollutants are usually detected in combination, a binary mixture (MIX) of DCF and SMX was prepared. The synthetic domestic wastewater was prepared in tap water according to a modified protocol previously described by Nopens et al. [31] (Appendix A—Table A2). Wastewater samples were collected within two months after obtaining the stable operation of the systems. Each sample was analyzed separately in three technical replicates. The types of columns used in this experimental system, with assigned symbols, are listed in Table 2.

### 2.3. Chemical Analysis of Wastewater

The measured chemical parameters included DCF and SMX concentration, ammonium nitrogen (N-NH_4_, mg L^−1^) and total organic carbon (TOC, mg L^−1^) concentration. Previous study had indicated that these parameters had the highest impact on the wastewater treatment process [32]. The procedure used for analysis is described in Appendix A, Appendix A.1.3.

### 2.4. Activity of Antioxidant Enzymes in M. giganteus

For plant material enzymatic assays, a Pro200 homogenizer (Pro Scientific Inc., Oxford, CT, USA) was used to prepare leaf homogenates. Three similar-sized leaves of *M. giganteus* were taken from each test column after 241–290 days of experiment. For determination of catalase (CAT) activity, 0.06 M sodium phosphate buffer (pH 7.4) was added to homogenates, while 0.05 M carbonate buffer (pH 10.2) was added to determine superoxide dismutase (SOD) activity. CAT activity was determined using a static method described by Góth [33], while SOD activity was measured using method described by Misra and Fridovich [34]. The homogenates were centrifuged (20 min, 4000 rpm, 4 °C) and then stored at −45 °C until the antioxidant enzyme activity assays were performed.

Measurement of protein quantity and enzymatic activity in the samples was performed using an Evolution 220 spectrophotometer (Thermo Fisher Scientific, Waltham MA, USA) and Insight 2 software (2014) (Thermo Fisher Scientific, Waltham, MA, USA). Before measuring CAT and SOD enzymatic activity, the protein content in the samples was determined using the Bradford method [35]. The protein concentration of each prepared extract was determined with reference to a standard curve, using bovine albumin as the standard protein. The detail procedure used for enzymatic analysis is described in Appendix A, Appendix A.1.4.

### 2.5. Toxicity Tests towards Aquatic Organisms

Toxicity of samples towards *A. fischeri* (Modern Water, York, UK) was determined according to ISO 11348-3:2007 [36] using synthetic sea water, prepared in accordance with ISO 10253:2016 standards, as a control solution [37]. Microelements present in municipal wastewater (MgCl_2_, Na_2_SO_4_, CaCl_2_ or KCl) intensify the activity of microorganisms; therefore, a stimulatory response may be observed. The use of synthetic seawater eliminated the hormetic effect and increased bacterial sensitivity to the tested substances [38]. The composition of synthetic sea water is presented in Appendix A—Table A3. Inhibition of *A. fischeri* luminescence was determined (Microtox M500, Modern Water) after 15 min incubation and each assay was performed in 3 replicates.

Toxicity of samples toward *D. magna* was determined using the Daphtoxkit F Magna kit (Microbiotest Inc., Gent, Belgium). The toxicity tests were performed in accordance with OECD 202 [39]. The tests were performed in triplicate and the effect concentrations (EC_50_) were determined using logarithmic-probit and regression analysis methods.

The *L. minor* toxicity test was performed in accordance with OECD 221 [40] procedure in 3 replicates. After 7 days of incubation, the number of fronds was counted, and based on these data, the average growth rate (*μ_i−j_*) and the percentage inhibition of average growth rate (% *I_r_*) for each solution were calculated based on Equations (1) and (2), respectively:

(1) $$\mu_{i-j}=\frac{ln\left(N_{j}\right)-ln\left(N_{i}\right)}{t}\;$$

(2) $$\%\;I_{r}=\frac{\mu_{C}-\mu_{T}}{\mu_{C}}\;\cdot 100\;$$

where *N_i_*_(*j*)_ is the number of fronds in time *i* (*j*); t is the time between *i* and *j*; *μ_C_* is the mean *μ* value for the control solution; and *μ_T_* is the mean *μ* value for the tested concentration.

### 2.6. Toxicity Classification of Wastewater

The toxic unit (*TU*) value classification [41] was used to determine the toxicity of wastewater. The *TU* values for each test organism were calculated as per Equation (3):

(3) $$TU=\frac{1}{EC_{50}\left(IC_{50}\right)}\;\cdot 100\;$$

The classification of wastewater according to the *TU* value is presented in Appendix A—Table A4. When the toxic effect of the undiluted sample of wastewater was in the range of 10–49%, the *TU* value was calculated based on Equation (4) [42]:

(4) TU=0.02 ·E 

where *E* is the toxic effect of the undiluted sample of wastewater on the test organism.

If the toxic effect of the undiluted sample of wastewater is lower than 10%, the *TU* takes the value 0 [41].

### 2.7. Statistical Analysis

Statistical tests were performed using STATISTICA 13 software (StatSoft Poland, Kraków, Poland). Statistical tests in environmental studies are an indispensable part of the analysis of results, since we usually have to deal with natural variability in results related to, for example, genetic variation or physiological fluctuations. The first step in the statistical analysis was to verify the normal distribution of the results obtained. This is an important step, as it allows for the selection of an appropriate statistical test for assessing the significance of differences between variants of the experiment. In the case of our study, we demonstrated the significance of differences between the effects induced by control and pharmaceutical-containing wastewater, as well as verified whether differences in the way that R1 and R2 columns are fed have a significant effect on the effect of pollutant removal, toxicity of treated wastewater and enzyme activity in wetland plants (*Miscantus giganteus*). Normality of the data was tested using the Shapiro–Wilk test. For data sets with normal distribution, Student’s *t*-tests were used (α = 0.05). Otherwise, the data were analyzed using the Mann–Whitney U test. The differences were considered statistically significant if *p* < 0.05. The Kolmogorov–Smirnov (K-S) test (α = 0.025) was used to analyze the calculated HC_5_ values.

## 3. Results

### 3.1. Chemical Analysis of Wastewater

Wastewater treatment was assessed based on standard chemical parameters such as TOC (Appendix A—Figure A1) and N-NH_4_ concentration (Appendix A—Figure A2), as well as on efficiency of removal of pharmaceutical water contaminants (Appendix A—Figure A3). These parameters were determined both in the inflow and outflow from the CWs. The achieved removal efficiency of chemical parameters is presented in Table 3.

More than 87% of TOC was removed in all test columns (Table 3). However, the feeding frequency had an impact on the rate of TOC removal by the CWs. Higher removal rated were observed for R2 columns (feeding regime of 5 times a week in a volume of 1 L) with lower HLR. The presence of *M. giganteus* in the test columns did not have a significant effect on TOC removal. *M. giganteus* is a plant used as an energy crop and is characterized by rapid growth, even in poor soils. It can also be used in the CW wastewater treatment process [43]. In the case of N-NH_4_, the removal rate ranged from 18.0 to 58.9% (Appendix A—Figure A2; Table 3), and the presence of plants also did not have a statistically significant impact on the removal of this parameter. However, the removal of N-NH_4_ was influenced by the frequency of wastewater dosing and the presence of pharmaceuticals. More efficient removal of TOC and N-NH_4_ was observed from wastewater applied to R2 columns (feeding frequency of 5 times a week in a volume of 1 L) (average removal for TOC = 93.4% and for N-NH_4_ = 48.0%) compared with their removal from wastewater dispensed to R1 columns (feeding frequency of twice per week at a volume of 2.5 L) (89.0% and 25.0%, respectively). Thus, it could be seen that the frequency of wastewater dosing had the greatest impact on the removal of both organic and nitrogen compounds.

DCF removal efficiency in all analyzed samples was lower than SMX removal (Appendix A—Figure A3; Table 3). The frequency of wastewater dosing was found to affect the removal efficiency of DCF and SMX. Better removal of both pharmaceuticals was observed in the effluent from R2 columns compared to the effluent from R1 columns, which was confirmed by statistical analyses (Appendix A—Figure A3). It was observed that the presence of *M. giganteus* in CW influenced the removal of DCF in all columns tested, while in the case of SMX, the presence of plants influenced its removal only in R2 columns.

### 3.2. Antioxidant Enzymes in M. giganteus

**Figure 3:**
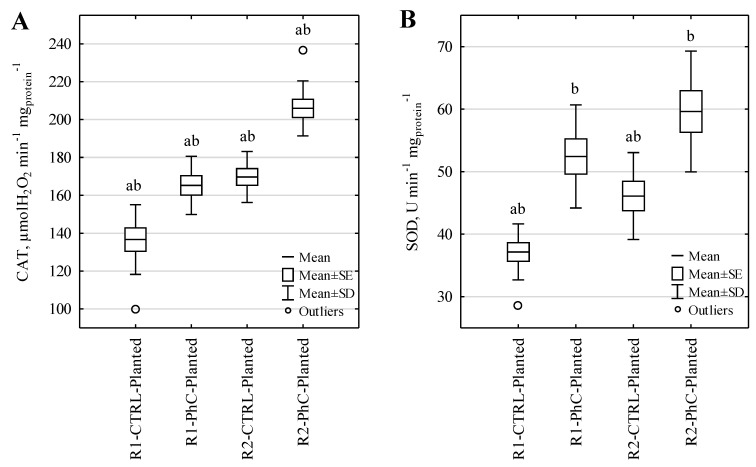
Activity of CAT (**A**) and SOD (**B**) in *M. giganteus*. (a) Statistically significant differences between counterpart types of columns (CTRL or PhC) with different frequency of wastewater dosing (Student’s *t*-test, α = 0.05); (b) statistically significant differences between control wastewater and wastewater containing pharmaceuticals (Student’s *t*-test, α = 0.05).

The activity of CAT and SOD in *M. giganteus* are presented in Figure 3. The effect of the presence of pharmaceuticals and the frequency of wastewater dosing on the antioxidant enzymes’ activity in *M. giganteus* plants was observed (Figure 3, Appendix A—Table A5). Higher activity of these enzymes was noted in plants from R2 columns (feeding frequency of 5 times a week in a volume of 1 L). CAT activity in plants from R1 columns fed with control wastewater averaged 136.7 µmolH_2_O_2_ min^−1^ mg_protein_^−1^, while that in plants from R2 columns fed with the same type of wastewater averaged 169.7 µmolH_2_O_2_ min^−1^ mg_protein_^−1^. A similar phenomenon was observed for columns fed with wastewater containing pharmaceuticals. In plants from R1 columns, the average CAT activity was 165.3 µmolH_2_O_2_ min^−1^ mg_protein_^−1^, while an increase in CAT activity was also observed in plants from R2 columns, with an average of 205.9 µmolH_2_O_2_ min^−1^ mg_protein_^−1^. As the statistical analyses showed, the differences between CAT activity in plants from R1 and R2 columns were statistically significant with α = 0.05.

In the case of SOD, an increase in the activity of this enzyme was observed in leaf samples taken from R2 columns compared to leaf samples taken from R1 columns. For samples taken from columns fed with control wastewater, the average activity of SOD was 37.2 U min^−1^ mg_protein_^−1^ for columns from the R1 system and 46.1 U min^−1^ mg_protein_^−1^ for columns from the R2 system. Statistical analysis showed that for the control wastewater, the difference in SOD activity between R1 and R2 was statistically significant, with α = 0.05.

For leaf samples taken from columns fed with pharmaceutical-containing wastewater, SOD activity was higher than for samples taken from columns fed with control wastewater (R1: average SOD activity 52.4 U min^−1^ mg_protein_^−1^, R2: average SOD activity 59.6 U min^−1^ mg_protein_^−1^). The observed differences were statistically significant only in comparison to the control wastewater.

Differences in SOD activity in leaf samples taken from R1 and R2 columns fed with pharmaceutical-containing wastewater were statistically insignificant. We believe that the method of feeding influences SOD activity (which was proven in the case of control wastewater), but the introduction of an additional stress factor, such as the presence of pharmaceuticals, masked this effect. The likely reason for the lack of statistically significant differences in the case of SOD is the masking of the toxic effect by a combination of stressors. It is also worth noting that the presence of CAT will be a more sensitive indicator of oxidative stress in *M. giganteus*. The main reason for the observed dissimilarity between the enzymes activity under stress conditions is the different mechanism of action of CAT and SOD. This was the first observation of this kind of relationship between operating parameters of CWs and activity of antioxidant enzymes in *M. giganteus*.

### 3.3. Toxicity of Wastewater

**Figure 4:**
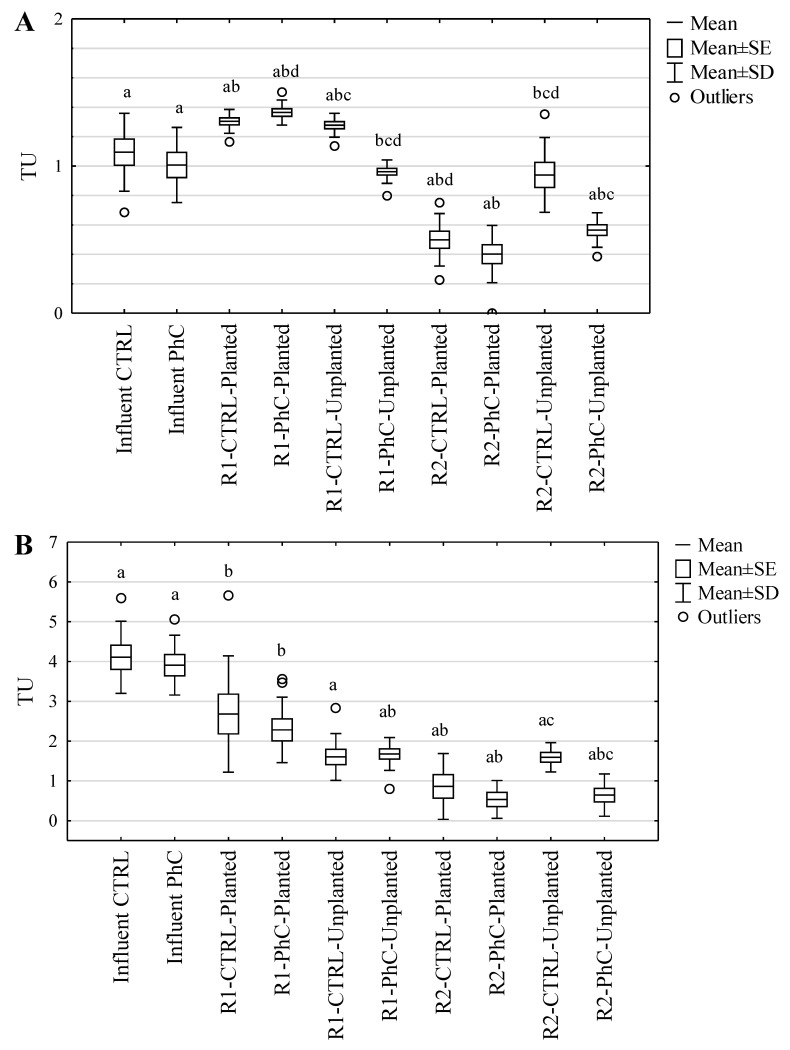

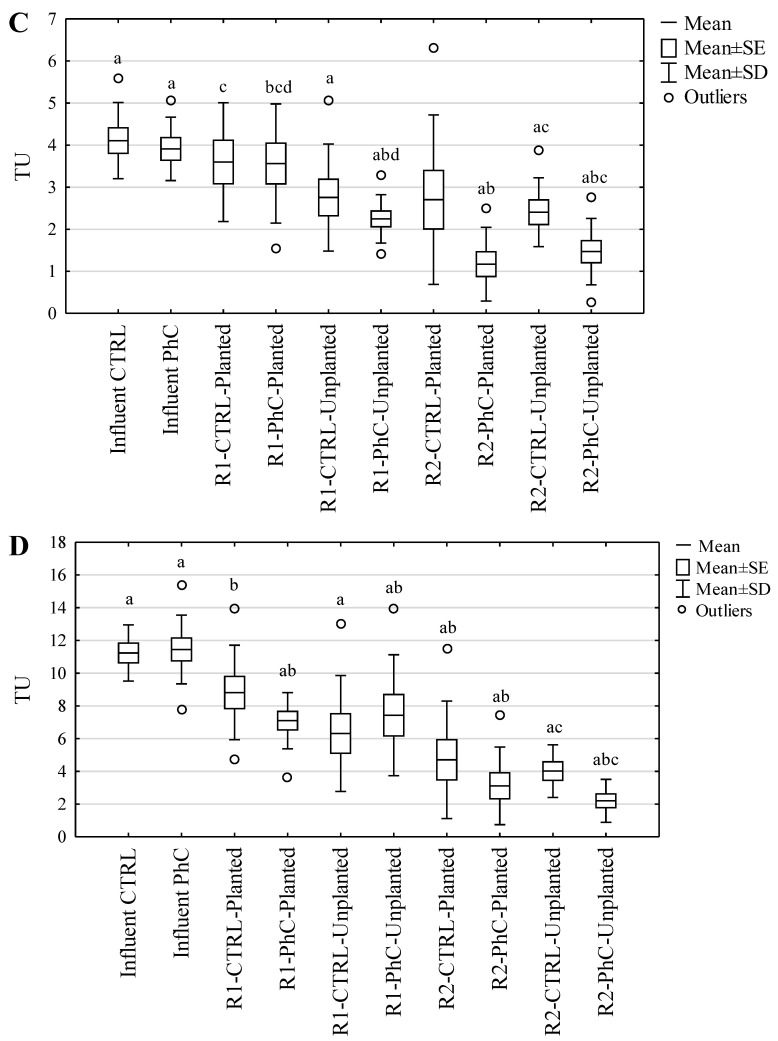
Toxicity of wastewater to aquatic organisms: (**A**) *A. fischeri*; (**B**) *D. magna*, 24 h; (**C**) *D. magna*, 48 h; (**D**) *L. minor*. (a) Statistically significant differences between the wastewater influent and effluent (Student’s *t*-test, α = 0.05); (b) statistically significant differences between counterpart types of columns (CTRL or PhC) with different frequency of wastewater dosing (Student’s *t*-test, α = 0.05); (c) statistically significant differences between control wastewater and wastewater containing pharmaceuticals (Student’s *t*-test, α = 0.05); (d) statistically significant differences between wastewater from planted columns and from columns without plants (Student’s *t*-test, α = 0.05).

In Figure 4, the TU values, indicating toxicity of the wastewater toward *A. fischeri*, *D. magna* and *L. minor*, are shown. A decrease was observed in wastewater toxicity after treatment toward all organisms except *A. fischeri*.

Based on these data, the average wastewater toxicity reduction (WTR, %) was calculated according to Equation (5) and is presented in Appendix A—Table A6.

(5) $$WTR=\frac{\left(TU_{IN}-TU_{EF}\right)\cdot 100\;}{TU_{IN}}\;$$

where *TU_IN_* is the average toxic unit calculated for influent wastewater and *TU_EF_* is the average toxic unit calculated for effluent wastewater.

Wastewater toxicity reduction (Appendix A—Table A6) was higher for R2 columns (average WTRs toward *A. fischeri* and *D. magna* after 48 hrs and *L. minor* were 43.8, 52.0 and 73.9%, respectively) in comparison to R1 columns (average WTRs toward test organisms were 22.3, 24.2 and 40.9%, respectively). The differences in wastewater toxicity reduction were associated with wastewater feeding frequency.

Based on the toxicity classification of wastewater (Table 4), a reduction in toxicity class from high (for influent) to average (for effluent) can be observed and was validated by statistical analyses (Mann–Whitney U test, *p* < 0.05, Table 4).

To determine the hazardous concentration of wastewater for 5% of species, HC_5_ values were calculated using a species sensitivity distribution model [44,45]. The HC_5_ values and 95% confidence limits, including lower limit (LL) and upper limit (UL), are shown in Table 5. All samples met the normal distribution criteria for the Kolmogorov–Smirnov (K-S) test (α = 0.025). The analyses represent *n* = 16 repeats, and the critical value of K-S statistics was 0.995. The NOEC parameter was used as a baseline to calculate HC_5_, denoting the highest concentration with no observed harmful effect on model organisms. Low HC_5_ values indicate that contamination of surface water with the analyzed wastewater can cause harmful effects to organisms living in this environment. Even slight contamination of the environment with raw wastewater possessing a low HC_5_ value can be dangerous for living organisms (determined HC_5_ at the level of 5.7%). Treated wastewater demonstrated less harmful ecotoxicological properties, with HC_5_ values for the effluents ranging from 15.8–24.0%.

## 4. Discussion

### 4.1. Chemical Parameters of Treatment Process

The results showed that the effect of the feeding frequency affected all determined chemical parameters. In the case of TOC concentration, higher removal rates were observed for R2 columns (feeding regime of 5 times a week in a volume of 1 L). Similar relationships were reported by Saeed and Sun [46], who showed that the dosing regime and the type of CWs used can affect the removal efficiency of organic compounds. These findings are reflected in the literature concerning vertical-flow CWs [47,48]. The mode of CW operation can impact the oxidation and reduction processes occurring in the system and the diffusion of oxygen into the bed. Periodic dosing that provides oxygen conditions can result in better removal efficiency than continuous feeding [49]. However, the denitrification process can be limited in such systems [50,51]. For planted columns, improved N-NH_4_ removal from wastewater may be related to uptake by plants and stimulation of rhizosphere bacteria rhizosphere to oxidize ammonium nitrogen [52].

The presence of plants in CWs may therefore play a role in the removal of some pharmaceuticals, such as DCF. Plants can take up contaminants but also stimulate development of bacteria in the rhizosphere [49]. In addition, the presence of plants can affect the pharmaceuticals’ removal through root exudates. The release of some plants’ metabolites, such as threonine, citronellic acid or acrylic acid derivate, was combined to higher PhC removal in the columns with *Phragmites australis* plants [53].

The uptake of xenobiotics by plants is an important process contributing to the removal of micropollutants. This process mainly depends on the sorption of pollutants onto soil particles, as the primary substrate of the CW system. Only contaminants that are in the pore water can be taken up by the root system. Sorption also affects the availability of PPCPs to microorganisms, and consequently, the effectiveness of microbial degradation [54]. Diffusive translocation of pharmaceuticals into plant tissues depends mainly on the physicochemical properties of these compounds. The most important parameters are water solubility and hydrophobicity, characterized in terms of the octanol:water partition coefficient, log K_OW,_ of the compound [55]. Moderate hydrophobicity, as indicated by a log K_OW_ in the range of 0.5–3.5, is considered optimal for a compound to be taken up by plants [56]. Chemical compounds with a log K_OW_ above 3.5 (such as DCF, Appendix A—Table A1) are less likely to diffuse into the roots of plants; for this reason, the bacteria from the rhizosphere are required to be more involved in DCF removal [55,56]. PPCPs can be removed by plants in the CW directly, as described above, or indirectly. The indirect effect of plants in removing xenobiotics is mainly to promote the growth and activity of rhizosphere microorganisms through the plant root system. In addition, each plant species used in CW has a specific population of microorganisms in the rhizosphere, and thus the distribution of pollutants, including PPCPs, may differ [57]. In our study, the presence of *M. giganteus* in CWs was not significant for the removal of pharmaceuticals, but in earlier work we showed a positive effect of another plant, *Phragmites australis*, on the removal of pharmaceuticals [30].

The designed technological parameters also have a significant impact on the efficiency of wastewater treatment in CWs. For submerged wetlands, periodic aeration of the bed is recommended to ensure aerobic conditions, providing better removal of carbon and nitrogen compounds. In addition, the use of plants helps transfer oxygen to the root portion of plants located in CW beds [58]. The composition of the bed, which is involved in the filtration or absorption of contaminants, among other things, is also an important element affecting the efficiency of CWs [59].

For full-scale CWs, the climatic variable will also affect the efficiency of contaminant removal. In the case of our laboratory-scale CWs, this impact has been minimized by controlling basic parameters (such as temperature, light intensity, length of day and night). However, the impact of these parameters on CW performance should be investigated and requires further validation. Comparing the results of DCF and SMX removal in CWs with those reported by other researchers, it can be concluded that the results we obtained are average for biological systems. The biodegradation process leads to only moderate removal of active pharmaceutical substances [30,60,61,62].

In the literature, one can find information about unconventional studies related to the removal of SMX from wastewater. For example, the degradation of SMX by microalgae was studied [63]. The observed maximum biodegradation of SMX was 99.3% and was achieved by a pure culture of *Chlorella pyrenoidosa* with initial SMX concentration of 0.1 mg L^−1^ [64]. In contrast, the removal efficiency of SMX in the bacterial–microalgae hybrid system was lower, with the maximum elimination reaching 40.84 ±6.0% when the initial SMX concentration was 1 mg L^−1^ [63]. Thus, in our study, pharmaceutical removal was at a satisfactory level, especially considering the low operating costs.

### 4.2. Activity of M. giganteus Enzymes

An important factor impacting the use of plants in CWs is potential toxicity of the wastewater. Oxidative stress, a state of imbalance between the production of reactive oxygen species (ROS) and their elimination by antioxidant systems, is one of the measures of harmfulness of wastewater to organisms. Many substances present in the environment can cause increased ROS production and resultant oxidative stress, leading to negative effects on living organisms [65]. Factors that can affect oxidative stress include temperature, oxygenation, salinity, metal ions, pesticides and other chemical contaminants [65]. In a healthy organism, there should be a balance between ROS production and the protective antioxidant systems and organisms have developed a series of defense mechanisms against ROS. These mechanisms include prevention of excessive ROS formation, termination of radical reactions and removal of the effects of ROS by means of antioxidant enzymes that are designed to remove excess reactive molecules [66].

The antioxidant enzymes CAT and SOD are responsible for reducing oxidative stress and play a very important role in plant survival [67]. In plant cells, SOD can be divided into three groups: Fe SOD (located in chloroplasts), Mn SOD (in mitochondria and peroxisomes) and Cu-Zn SOD (located in chloroplasts and cytosol). The action of SOD represents the most important defense mechanism in plants and crucial for the detoxification process [68].

The activity of antioxidant enzymes is often used to gauge the effect that impurities have on plants used in CWs [47,68,69]. Elevated CAT and SOD activity may result from extended contact of *M. giganteus* with the wastewater being analyzed, since the leaves used for enzymatic assays were collected on 241–290 days after first exposure of plants to wastewater. Yan et al. [70] similarly suggested that an increase in antioxidant activity is associated with an increase in the contact time of plants with wastewater and also showed that plants can take up and potentially metabolize pharmaceuticals.

Catalase is considered to be a bioindicator of oxidative stress from various pollutants. In *Oriza sativa* plants, an increase in the activity of this enzyme was observed under the influence of wastewater from a rice mill in pot culture [71]. In addition, in the case of plant contact with heavy metals, a relationship was observed between the concentration of the tested substances and the activity of CAT and SOD. The increase in catalase activity was correlated with the concentration of heavy metals in the *Typha latifolia* leaves, but the rhizomes of the same plants did not show increased catalase activity [72]. In our study, we observed an increase in CAT and SOD activity *in M. giganteus* leaf samples taken from racks fed with wastewater containing DCF and SMX. We observed an increase in CAT activity of 120% for R1 columns and 121% for R2 columns with respect to the enzyme activity in *M. giganteus* leaves watered with control (synthetic wastewater). As for SOD activity, an increase of 141% and 129% was observed for Rack 1 and Rack 2, respectively.

The activity of antioxidant enzymes is a very useful tool for assessing the long-term harmfulness of tested substances to aquatic plants. An increase in enzyme activity indicates the onset of oxidative stress, and therefore a disturbance of homeostasis in the plant. By assessing enzyme activity, we can detect changes before visible damage occurs (e.g., wilting of leaves, stunting of plant growth). It would be a very valuable study to determine the activity of antioxidant enzymes at multiple time points during the experiment. This would give a picture of the changes that occur in plants during prolonged exposure to contaminants.

### 4.3. Toxicity of Wastewater

The operating parameters of the CWs affected the treated wastewater and thereby the effectiveness of treatment process. However, the removal of organic carbon, nitrogen compounds or xenobiotics cannot be treated as synonymous with wastewater toxicity reduction.

In the case of the *A. fischeri*, a reduction in the toxicity of wastewater was observed after the treatment by all types of columns with lower feeding frequency (R2) and by R1-PhC-Unplanted columns. An increase in toxicity observed in wastewater from the R1 columns (feeding frequency of twice per week at a volume of 2.5 L) might be associated with the generation of more toxic decomposition products [73] or slower distribution of primary metabolites. Sulfonamide antibiotics have been shown to undergo transformation in aqueous environments by biodegradation, photolysis or hydrolysis. Majewsky and coauthors [74] found that some SMX transformation products, especially 4-hydroxy-SMX and N4-hydroxy-acetyl-SMX, exhibit antimicrobial activity. The antimicrobial activity was tested for luminescence inhibition on *Vibrio fischeri*. It seems that in our study we observed exactly the same phenomenon of persistence of antimicrobial activity of SMX derivatives. This is also confirmed by our previous studies [30], in which we demonstrated the presence of SMX degradation products in CW effluents; these were the products of (mono-, di-, tri-)hydroxylation, demethylation and deamination reactions. The above examples indicate that even partially or completely transformed compounds (even if the parent compounds are not detectable in the environment) can cause similar or even greater effects. Such an effect is related to the pseudo-persistence of the antimicrobial agents [20].

It should also be kept in mind that wastewater is a cocktail of different substances that undergoes transformations during treatment that result in the formation of new compounds with different properties. Usually, the treatment process also results in a reduction in possible toxicity, but this is not always the case. For example, Punzi et al. [75] observed that textile wastewater showed higher toxicity to *V. fischeri* after anaerobic treatment. The introduction of an ozonation process significantly reduced the harmfulness of wastewater, indicating that the availability of oxygen may be a key element in the removal of contaminants from wastewater [75]. Our results indicate that the frequency of dosing of all wastewater samples affected toxicity to *A. fischeri* and *L. minor*. In the case of *D. magna*, the method of feeding affected toxicity only in wastewater samples containing pharmaceuticals. It was found that the toxicity of wastewater treated in R2 columns, dosed at 5 doses per week in a volume of 1 L per dose, was lower than that of wastewater treated in R1 columns: two doses per week in a volume of 2.5 L per dose. The literature on the frequency of wastewater dosing and its effect on wastewater toxicity is sparse. Previously, it was observed that the frequency of wastewater dosing had a significant effect on the removal of DCF and SMX in the same experimental system, but operating under different experimental conditions [30]. The toxicity of wastewater to *V. fischeri* was also partially influenced by the feeding regime used. Ávila et al. [50] analyzed the effect of HLR on leachate toxicity in hybrid artificial CWs. The experimental system was operated at HLRs of 0.06, 0.13 and 0.18 m^3^ m^−2^ d^−1^. The authors showed that in continuous feeding mode, high HLRs were able to reduce overall effluent toxicity by 90%.The presence of plants was shown to affect wastewater toxicity only toward *A. fischeri* and *D. magna. A. fischeri* luminescence was inhibited only by wastewater from R1-PhC and R2-CTRL columns (Figure 4A). The *D. magna* immobilization assay (Figure 4B,C) yielded statistically significant differences in toxicity between wastewater from planted and unplanted columns after 48 hrs of incubation with effluent from R1-PhC columns (Student’s *t*-test, α = 0.05). The presence of *M. giganteus* plants in tested columns did not have a statistically significant effect on *L. minor* growth inhibition (Figure 4D). It had been previously observed that the toxicity of wastewater was 5–10% lower in columns that were not planted with *Phalaris arundinacea* [30]. This observation was associated with the dissolved organic carbon concentration. It has also been suggested that plant excretions may affect the toxicity of effluents from CWs. Root systems release various organic substances such as anaerobic metabolites, organic acids, steroids and even antimicrobial compounds. In addition, they may also secrete phytosiderophores, compounds that chelate iron, zinc, copper and manganese ions, which can affect the wastewater treatment process [76].

These findings suggest that DCF and SMX contents in wastewater are not the primary determinants of toxicity toward bioindicators as have been suggested by preliminary tests [77]. A statistically significant effect of pharmaceutical content on toxicity towards *A. fischeri* (Student’s *t*-test, α = 0.05) was observed only in wastewater from unplanted columns. Toxicity of pharmaceutical-containing wastewater towards *L. minor* was observed only with regard to wastewater from R2-Unplanted columns, and toward *D. magna* (after 48 hrs of incubation) by wastewater from R1-Planted and R2-Unplanted columns.

The hazardous concentration for 5% of species was calculated (Table 5) to assess the risks that raw and treated wastewater may enter into the environment. The HC_5_ value means the concentration of the substance, in this case wastewater, which is safe for 95% of species [44]. It is a worldwide problem that treated effluents constitute a considerable fraction of water in receiving waters under drought conditions as an effect of the climate change [78,79]. For this reason, the use of appropriate dilution factors can be crucial for environmental protection. Moreover, environmental samples are a mixture of different materials, and their exact composition is unknown and changes over time. For this reason, determining the harmful concentration value toward 5% of species may be valuable in assessing the purification process, even in the absence of complete information regarding wastewater composition.

In summary, wastewater from R2 columns was less harmful to aquatic bioindicators than the effluent from R1 columns. Toxicity tests showed that the frequency of dosing had a significant impact on the ecotoxicological properties of wastewater. The predominance of aerobic processes in columns with lower feeding frequency (R2) (wastewater dispensed 5 times a week in a volume of 1 L) provides better conditions for removing organic compounds, N-NH_4_ and pharmaceuticals, and resulted in superior overall detoxification of effluents.

It is also worth mentioning that the influence of TOC and N-NH_4_ on the toxicity of wastewater was observed. It was found that there was correlation between TOC and the toxicity of samples. It was also observed that higher correlation coefficients were obtained when results were divided into two groups: samples with TOC < 100 mg L^−1^ (which corresponds to treated wastewater) and samples with TOC ≥ 100 mg L^−1^ (raw wastewater). For each bioindicator, the correlation between toxicity, TOC and N-NH_4_ (investigated as related parameters) was determined, while also identifying parameters for which wastewater was nontoxic to organisms. Appendix A—Figure A4 provides an example of the correlations between the analyzed parameters and wastewater toxicity toward to the bioindicator *D. magna*. The calculated correlations presented in Appendix A—Figure A4 are relevant to wastewater samples that comprise the general properties of household wastewaters and do not contain additional specific toxic compounds aside from pharmaceuticals.

The equations obtained from correlation analysis between the chemical parameters of wastewater and their toxicity are presented in Appendix A—Table A7. According to the Persoone et al. [41] classification, the TU value for wastewater below 0.4 is regarded as safe for the environment. Since TOC and N-NH_4_ tolerance have been analyzed in combination, if the concentration of organic compounds in the sample is very low (near zero), then model organisms may tolerate higher concentrations of N-NH_4_ (up to 26 mg L^−1^) while maintaining the same TU toxicity level of 0.4. When the N-NH_4_ concentration tends to zero, the bioindicators’ tolerance to TOC concentration is approximately 35 mg L^−1^. When TOC is above 100 mg L^−1^ (raw wastewater), wastewater toxicity is very high (exhibiting a mean TU > 2) even if N-NH_4_ concentration tends to zero. This influence of primary contaminants, expressed as TOC and N-NH_4_ concentration, on the ecotoxicological properties of wastewater is an important observation with relevance to future long-term experiments involving wastewater of known composition.

Detoxification, according to the definition proposed by Fortney et al. [80], is a set of processes whose purpose is to identify, neutralize and eliminate toxic substances and their degradation products. This definition can refer to both the medical and environmental aspects of this process. Therefore, detoxification can be defined as a process of reducing the toxicity of a substance or wastewater to an environmentally safe level. According to the above definition, detoxification of wastewater was not achieved during the present study, as all effluent samples from CWs exhibited average toxicity to aquatic bioindicator organisms; however, it was confirmed that ecotoxicity testing of wastewater provides a broader and deeper insight to the efficacy of wastewater treatment processes. Ecotoxicological analysis of wastewater is not required in the majority of European countries, where the assessment of wastewater treatment is typically limited to the analysis of physicochemical parameters. Treated wastewater must be characterized by an appropriate level of contaminants or a sufficiently high removal efficiency of a given parameter before being introduced into the natural environment [17]. The data presented here emphasize that supplementing physicochemical analyses with ecotoxicological tests is very valuable for improved environmental protection.

## 5. Conclusions

The conducted research showed that the use of bioindication methods complements the information obtained from standard physicochemical analysis. Only both types of results give a full picture of the threat posed by the analyzed pollutants to the natural environment. The ecotoxicological analysis provides information on the harmfulness of pollutants in the environment. The physicochemical analysis allows for only the assessment of the effectiveness of the wastewater treatment process; however, the by-products generated during the purification process may often show higher biological activity than the parent product. Therefore, discharge of treated wastewater into the environment might pose a serious risk to living organisms. The use of both analyses allows for a complete evaluation of the treated wastewater properties.

The reduction in wastewater toxicity was observed in the laboratory model of the vertical-flow CWs; however, detoxification of wastewater was not achieved, as confirmed by the HC_5_ values. Raw wastewater was characterized as highly toxic, while treated wastewater was classified as exhibiting average toxicity toward aquatic organisms. More frequent feeding resulted in improved ecotoxicological properties of wastewater effluents, which was assumed to be due to greater bed oxygenation. The presence of *M. giganteus* plants was not a determining factor of the efficacy of the purification process. However, it was observed for the first time that the antioxidant enzyme activity detected in *M. giganteus* leaves was influenced by the operating parameters of the CWs (wastewater dosing and the presence of pharmaceuticals).

## Figures and Tables

**Table 1:** Selected cost of constructed wetland and sequencing batch reactor operation [10].

Type of Costs	Unit	CWs	SBR
Electricity	KW year^−1^	260,000	7,500,000
Labor cost	Full-time equivalent year^−1^	0.75	12
Maintenance cost	DFC year^−1^	0.9	1.9
Miscellaneous cost	USD year^−1^	26,000	245,000
Impact on ozone depletion	kg CFC	6.6 × 10^−9^	6.6 × 10^−7^
Land requirement	m^2^ person^−1^	1.5–2.5	0.05–0.01

**Table 2:** Types of columns used in the experiment.

Types of Columns	R1-CTRL-Planted	R1-PhC-Planted	R1-CTRL-Unplanted	R1-PhC-Unplanted	R2-CTRL-Planted	R2-PhC-Planted	R2-CTRL-Unplanted	R2-PhC-Unplanted
Feeding regime (R)	R1				R2			
	2 days of feeding/5 days of resting				5 days of feeding/2 days of resting			
Wastewater volume	2.5 L/d 5 L/week				1 L/d 5 L/week			
HLR ^1^	0.08 m^3^ m^−2^ d^−1^				0.032 m^3^ m^−2^ d^−1^			
Plants	yes		no		Yes		no	
PhC ^2^	no	yes	no	yes	no	yes	no	yes

^1^ Hydraulic loading rate; ^2^ pharmaceuticals (DCF and SMX); CTRL columns—columns fed with synthetic wastewater; PhC columns—columns fed with synthetic wastewater containing DCF and SMX.

**Table 3:** Removal efficiency of TOC, N-NH_4_, DCF and SMX in CWs.

Types of Columns	Removal Efficiency, % *			
	TOC	N-NH_4_	DCF	SMX
R1-CTRL-Planted	87.8 ± 3.93	19.9 ± 7.3	–	–
R1-PhC-Planted	87.3 ± 1.94	18.0 ± 11.4	68.8 ± 8.2	79.1 ± 4.3
R1-CTRL-Unplanted	90.8 ± 1.08	39.3 ± 10.3	–	–
R1-PhC-Unplanted	90.0 ± 0.97	22.9 ± 12.6	13.7 ± 16.3	78.9 ± 22.5
R2-CTRL-Planted	93.3 ± 1.43	45.8 ± 11.9	–	–
R2-PhC-Planted	92.3 ± 1.12	58.9 ± 10.0	86.8 ± 9.7	98.0 ± 0.8
R2-CTRL-Unplanted	93.7 ± 1.07	29.1 ± 8.3	–	–
R2-PhC-Unplanted	94.1 ± 0.59	58.3 ± 4.6	76.6 ± 9.4	97.4 ± 0.7

* The removal efficiency (R) was calculated based on the influent and effluent concentrations according to $R\left(\%\right)=\frac{C_{influent}-C_{effluent}}{C_{influent}}\;\cdot 100$ .

**Table 4:** The TU value and toxicity classification of wastewater from CWs.

Types of Columns	TU				Toxicity Classification ^2^
	*A. fischeri* ^1^	*D. magna*, 24 h	*D. magna*, 48 h	*L. minor*	
Influent CTRL	1.1 ± 0.3 ^a^	3.1 ± 0.8 ^b^	4.1 ± 0.9 ^b^	11.2 ± 1.7 ^a^	high toxicity
Influent PhC	1.0 ± 0.3 ^a^	2.8 ± 1.0 ^b^	3.9 ± 0.8 ^b^	11.5 ± 2.1 ^a^	high toxicity
R1-CTRL-Planted	1.3 ± 0.1 ^a^	2.7 ± 1.5	3.6 ± 1.4	8.8 ± 2.9	average toxicity
R1-PhC-Planted	1.4 ± 0.1 ^a^	2.3 ± 0.8	3.6 ± 1.4	7.1 ± 1.7 ^a^	average toxicity
R1-CTRL-Unplanted	1.3 ± 0.1 ^a^	1.6 ± 0.6 ^b^	2.8 ± 1.3 ^b^	6.3 ± 3.5	average toxicity
R1-PhC-Unplanted	1.0 ± 0.1 ^a^	1.7 ± 0.4 ^b^	2.3 ± 0.6 ^b^	7.4 ± 3.7	average toxicity
R2-CTRL-Planted	0.5 ± 0.1 ^a^	0.9 ± 0.8 ^b^	2.7 ± 2.0 ^b^	4.7 ± 3.6 ^a^	average toxicity
R2-PhC-Planted	0.4 ± 0.2 ^a^	0.5 ± 0.8 ^b^	1.2 ± 0.9 ^b^	3.1 ± 2.4 ^a^	average toxicity
R2-CTRL-Unplanted	0.9 ± 0.3 ^a^	1.6 ± 0.4 ^b^	2.4 ± 0.8 ^b^	4.0 ± 1.6 ^a^	average toxicity
R2-PhC-Unplanted	0.6 ± 0.1 ^a^	0.6 ± 0.5 ^b^	1.5 ± 0.8 ^b^	2.2 ± 1.3 ^a^	average toxicity

^1^ [38]; ^2^ The toxicity data representing the most sensitive model organism determined the toxicity classification; (a) statistically significant differences between the wastewater influent and effluent in *A. fischeri* and *L. minor* tests (Student’s *t*-test, α = 0.05); (b) statistically significant differences between the wastewater influent and effluent in *D. magna* tests (Mann–Whitney U test, *p* < 0.05).

**Table 5:** HC_5_ values for wastewater from CWs.

Types of Columns	Parameter, %		
	LL ^1^	HC_5_	UL ^2^
Influent CTRL	4.0	5.7	7.2
Influent PhC	4.0	5.8	7.3
R1-CTRL-Planted	13.6	15.8	17.8
R1-PhC-Planted	14.0	16.4	18.5
R1-CTRL-Unplanted	13.9	17.2	20.7
R1-PhC-Unplanted	18.6	22.1	25.1
R2-CTRL-Planted	13.9	17.0	19.5
R2-PhC-Planted	16.0	18.5	20.8
R2-CTRL-Unplanted	18.1	21.5	23.1
R2-PhC-Unplanted	19.4	24.0	26.5

^1^ Lower limit; ^2^ upper limit.

**Figure A1:**
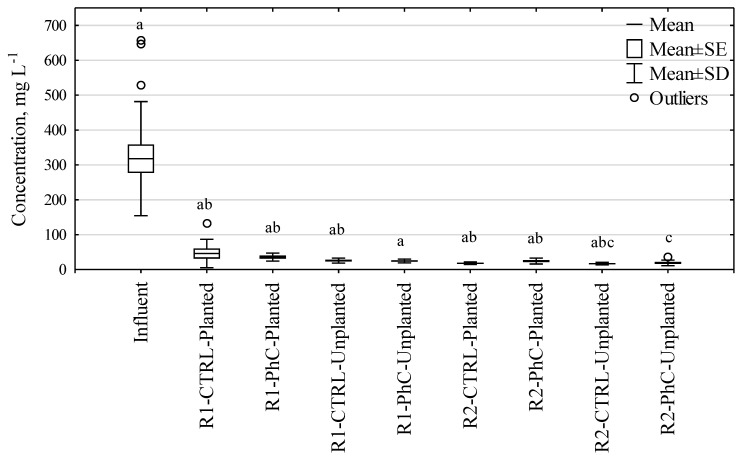
TOC concentration (mg L^−1^) of wastewater from CWs. (a) Statistically significant differences between the wastewater influent and effluent (Mann-Whitney U test, *p* < 0.05); (b) Statistically significant differences between counterpart types of columns (CTRL or PhC) with different frequencies of wastewater dosing (Mann-Whitney U test, *p* < 0.05); (c) Statistically significant differences between control wastewater and wastewater containing pharmaceuticals (Mann-Whitney U test, *p* < 0.05).

**Figure A2:**
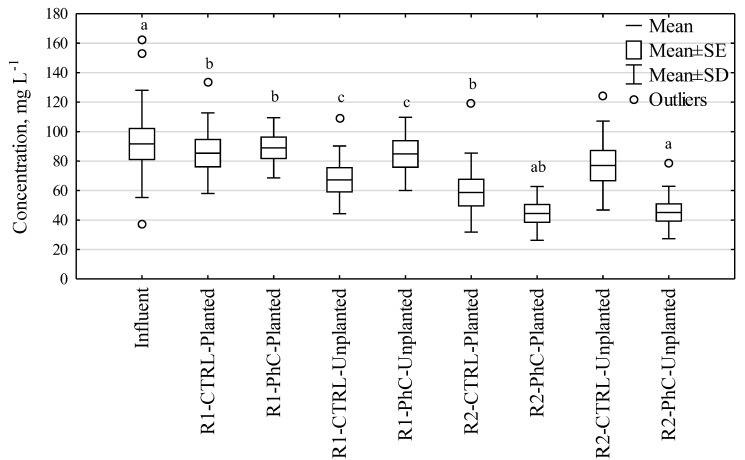
N-NH_4_ concentration (mg L^−1^) in wastewater from CWs. (a) Statistically significant differences between the wastewater influent and effluent (Mann-Whitney U test, *p* < 0.05); (b) Statistically significant differences between counterpart types of columns (CTRL or PhC) with different frequency of wastewater dosing (Mann-Whitney U test, *p* < 0.05); (c) Statistically significant differences between control wastewater and wastewater containing pharmaceuticals (Mann-Whitney U test, *p* < 0.05).

**Figure A3:**
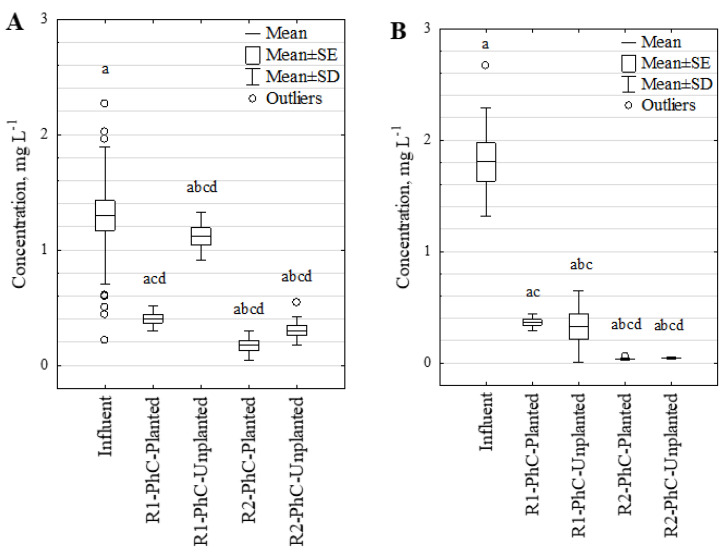
DCF (**A**) and SMX (**B**) concentration (mg L^−1^) in wastewater from CWs. (a) Statistically significant differences between the wastewater influent and effluent (Mann-Whitney U test, *p* < 0.05); (b) Statistically significant differences between DCF and SMX concentration (Mann-Whitney U test, *p* < 0.05); (c) Statistically significant differences between counterpart types of columns (CTRL or PhC) with different frequency of wastewater dosing (Mann-Whitney U test, *p* < 0.05); (d) Statistically significant differences between wastewater from planted columns and from columns without plants (Mann-Whitney U test, *p* < 0.05).

**Figure A4:**
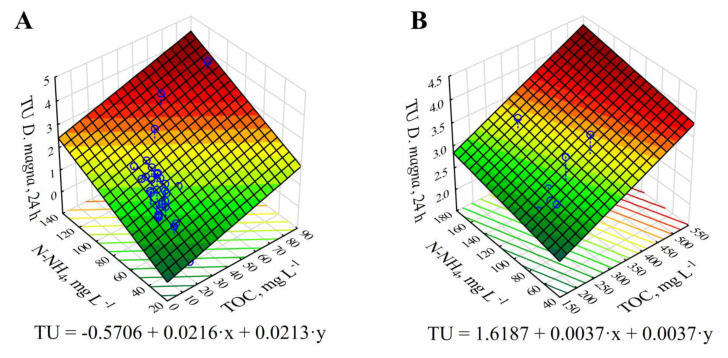
Correlations analysis between toxicity to *D. magna* and the chemical parameters of wastewater. (**A**) Wastewater with TOC < 100 mg L^−1^; (**B**) Wastewater with TOC ≥ 100 mg L^−1^; where x is the TOC concentration (mg L^−1^) and y is the N-NH_4_ concentration (mg L^−1^).

## Data Availability

Not applicable.

## Appendix A

### Appendix A.1. Materials and Methods

#### Appendix A.1.1. Tested Pharmaceuticals

**Table A1:** Basic properties and concentration of DCF and SMX in wastewater and in the environment.

Compound	DCF	SMX
CAS number	15307-86-5	723-46-6
Molecular formula	C_14_H_11_Cl_2_NO_2_	C_10_H_11_N_3_O_3_S
Molar mass, g mol^−1^	296.15	253.28
pK_a_ ^1^	4.15	5.6–5.7
log K_OW_ ^1^ (pH 8)	4.51	0.89
Maximal concentration in surface water ^2^, µg L^−1^	18.74	11.92
Maximal concentration in groundwater ^3^, µg L^−1^	0.59	1.11
Maximal concentration in wastewater effluent ^4,5^, µg L^−1^	5.5	6.0

^1^ [69] ^2^ [70] ^3^ [71] ^4^ [72] ^5^ [73].

#### Appendix A.1.2. Experimental System of CWs

**Table A2:** The recipe of synthetic municipal wastewater based on the modified protocol [28].

Chemical Compounds	Concentration, mg L^−1^	Food Ingredients	Concentration, mg L^−1^	Trace Metals	Concentration, mg L^−1^
CH_3_COONa	510.4	yeast extract	264	KCr(SO_4_)_2_∙12H_2_O	0.96
urea	208.76	skim milk powder	118	CuSO_4_∙5H_2_O	0.781
KH_2_PO_4_	41.37			NiSO_4_∙7H_2_O	0.359
peptone	40			ZnCl_2_	0.208
FeSO_4_∙7H_2_O	11.6			MnSO_4_∙H_2_O	0.108
MgSO_4_∙7H_2_O	4.408			PbCl_2_	0.1

All reagents were purchased from Avantor (Gliwice, Poland). The prepared synthetic wastewater was dosed to the surface of the bed, and then flowed vertically downwards until it reached the outlet zone.

#### Appendix A.1.3. Chemical Analysis of Wastewater

The wastewater samples (obtained by 8 sample collections) were filtered prior to further analysis. N-NH_4_ concentration was analyzed by means of an Ammonium Test kit (test no. 1.00683.0001, Merck, Germany). TOC was determined by TOC-L analyzer (Shimadzu Corporation, Kyoto, Japan).

Pharmaceuticals concentrations were monitored by HPLC using a C18 Hypersil^TM^ Gold column (250 mm × 4.6 mm, pore size: 5 μm; Thermo Scientific, Warsaw, Poland). The mobile phase consisted of a mixture of acetonitrile and acetate buffer (pH 5.7) in a volume ratio of 40:60 and was applied at an isocratic flow rate of 1.0 mL min^−1^. The retention time for DCF was 8.4 ± 0.3 min, and for SMX was 6.4 ± 0.2 min. The limit of quantification was 0.2 mg L^−1^. The tests were performed at four wavelengths: 220, 240, 268 and 280 nm. Data were analyzed using Chromeleon™ v. 6.8 software (Dionex Corporation, Sunnyvale, CA, USA).

#### Appendix A.1.4. Activity of Antioxidant Enzymes in *M. giganteus*

CAT activity (EC 1.11.1.6) was determined using a static method, measuring decomposition of hydrogen peroxide at a single time point, as described by [33]. The reaction was stopped after 4 min by adding 32.4 mM ammonium molybdate, which reacts with undecomposed H_2_O_2_ to form a colorimetric complex the intensity of which can be measured spectrophotometrically at 405 nm. A 65 mM solution of H_2_O_2_ in 0.06 M sodium phosphate buffer (pH 7.4) was used as the substrate. Enzyme activity is expressed in terms of μmol H_2_O_2_ min^−1^ mg_protein_^−1^.

SOD activity (EC 1.15.1.1) was measured using the self-oxidation reaction of adrenaline to adrenochrome at pH 10.2 as described by [34]. The measure of SOD activity is the inhibition of adrenaline oxidation over 4 min reaction time, using a 10 mM solution of adrenaline in 0.01 M HCl as reaction substrate. The amount of oxidized adrenaline is measured spectrophotometrically at 480 nm. SOD activity is expressed in terms of U min^−1^ mg_protein_^−1^. The arbitrary unit of activity (U) is defined as the amount of enzyme that leads to 50% inhibition of adrenaline oxidation within 1 min compared to auto-oxidation one would observe in a blank sample.

#### Appendix A.1.5. Toxicity Tests towards Aquatic Organisms

**Table A3:** Composition of synthetic sea water used as a control solutions in *A. fischeri* toxicity test.

Component	Concentration, g L^−1^
NaCl	22.0
MgCl_2_·6H_2_O	9.7
Na_2_SO_4_	3.7
CaCl_2_	1.0
KCl	0.65
NaHCO_3_	0.2
H_3_BO_3_	0.023

**Table A4:** The classification of wastewater according to the TU value [42].

TU Value	Toxicity Classification
<0.4	no toxicity
0.4–1	low toxicity
1–10	average toxicity
10–100	high toxicity
>100	extreme toxicity

### Appendix A.2. Results

According to Li et al. [55], pharmaceuticals removed via CWs can be classified as easily removable (removal rate > 70%), moderately removable (50–70%), not-easily removable (20–50%) and almost non-removable (removal rate < 20%). According to this classification, DCF may be classified as a moderately removable compound (on average 61.5% removal), whereas SMX was easily removable by CWs treatment (on average 88.4% removal).

**Table A5:** Activity of antioxidant enzymes in *M. giganteus*.

Types of Columns	Activity of Antioxidant Enzymes	
	CAT µmol H_2_O_2_ min^−1^ mg_protein_^−1^	SOD U min^−1^ mg_protein_^−1^
R1-CTRL-Planted	136.7 ± 18.4	37.2 ± 4.5
R1-PhC-Planted	165.3 ± 15.4	52.4 ± 8.2
R2-CTRL-Planted	169.7 ± 13.4	46.1 ± 6.9
R2-PhC-Planted	205.9 ± 14.5	59.6 ± 6.7

**Table A6:** Wastewater toxicity reduction as a result of treatment in CWs.

Types of Columns	The Wastewater Toxicity Reduction, %			
	*A. fischeri*	*D. magna*, 24 h	*D. magna*, 48 h	*L. minor*
R1-CTRL-Planted	−24.8	14.6	12.4	29.1
R1-PhC-Planted	−42.2	19.4	8.9	38.7
R1-CTRL-Unplanted	−22.5	48.9	32.9	54.2
R1-PhC-Unplanted	0.3	40.8	42.5	41.5
R2-CTRL-Planted	55.3	72.6	34.2	69.0
R2-PhC-Planted	62.1	82.6	70.1	78.3
R2-CTRL-Unplanted	14.3	49.2	41.4	65.4
R2-PhC-Unplanted	43.3	77.3	62.4	82.8

**Table A7:** Equations obtained from correlation analysis.

Test Organisms	TOC Concentration, mg L^−1^	
	<100 mg L^−1^	≥100 mg L^−1^
*A. fischeri*	TU = 0.1509 + 0.0029x + 0.01y	TU = 0.5195 + 0.0014x + 0.0015y
*D. magna*, 24 h	TU = −0.5706 + 0.0216x + 0.0213y	TU = 1.6187 + 0.0037x + 0.0037y
*D. magna*, 48 h	TU = −0.1875 + 0.0334x + 0.0266y	TU = 2.0676 + 0.0044x + 0.0075y
*L. minor*	TU = −1.4193 + 0.045x + 0.0803y	TU = 8.9237 + 0.01x − 0.0031y

Where: x is the TOC concentration (mg L^−1^), while y is the N-NH_4_ concentration (mg L^−1^).

## References

[1] Tang J., Zhang C., Shi X., Sun J., Cunningham J.A. (2019). Municipal wastewater treatment plants coupled with electrochemical, biological and bio-electrochemical technologies: Opportunities and challenge toward energy self-sufficiency. J. Environ. Manag..

[2] Wu H., Zhang J., Ngo H.H., Guo W., Hu Z., Liang S., Fan J., Liu H. (2015). A review on the sustainability of constructed wetlands for wastewater treatment: Design and operation. Bioresour. Technol..

[3] Doble M., Doble M., Kumar A. (2005). Treatment of waste from organic chemical industries. Biotreatment of Industrial Effluents.

[4] Liu L., Fan H., Huang X., Wei L., Liu C. (2019). Fate of antibiotics from swine wastewater in constructed wetlands with different flow configurations. Int. Biodeterior. Biodegrad..

[5] Bakhshoodeh R., Alavi N., Oldham C., Santos R.M., Babaei A.A., Vymazal J., Paydary P. (2020). Constructed wetlands for landfill leachate treatment: A review. Ecol. Eng..

[6] De Martis G., Mulas B., Malavasi V., Marignani M. (2016). Can artificial ecosystems enhance local biodiversity? The case of a constructed wetland in a Mediterranean urban context. Environ. Manag..

[7] Vymazal J. (2019). Is removal of organics and suspended solids in horizontal sub-surface flow constructed wetlands sustainable for twenty and more years?. Chem. Eng. J..

[8] Stanković D. (2017). Constructed wetlands for wastewater treatment. Građevinar.

[9] Zhi W., Ji G. (2012). Constructed wetlands, 1991–2011: A review of research development, current trends, and future directions. Sci. Total Environ..

[10] Parde D., Patwa A., Shukla A., Vijay R., Killedar D.J., Kumar R. (2021). A review of constructed wetland on type, technology and treatment of wastewater. Environ. Technol. Innov..

[11] Prado M., Borea L., Cesaro A., Liu H., Naddeo V., Belgiorno V., Ballesteros F. (2017). Removal of emerging contaminant and fouling control in membrane bioreactors by combined ozonation and sonolysis. Int. Biodeterior. Biodegrad..

[12] Chen W.-H., Wong Y.-T., Huang T.-H., Chen W.-H., Lin J.-G. (2019). Removals of pharmaceuticals in municipal wastewater using a staged anaerobic fluidized membrane bioreactor. Int. Biodeterior. Biodegrad..

[13] Meffe R., de Bustamante I. (2014). Emerging organic contaminants in surface water and groundwater: A first overview of the situation in Italy. Sci. Total Environ..

[14] Sousa J.C.G., Ribeiro A.R., Barbosa M.O., Pereira M.F.R., Silva A.M.T. (2018). A review on environmental monitoring of water organic pollutants identified by EU guidelines. J. Hazard. Mater..

[15] Yang Y., Ok Y.S., Kim K.-H., Kwon E.E., Tsang Y.F. (2017). Occurrences and removal of pharmaceuticals and personal care products (PPCPs) in drinking water and water/sewage treatment plants: A review. Sci. Total Environ..

[16] European Commission (2015). Commission Implementing Decision (EU) 2015/495 of 20 March 2015 Establishing a Watch List of Substances for Union-Wide Monitoring in the Field of Water Policy Pursuant to Directive 2008/105/EC of the European Parliament and of the Council.

[17] European Commission (2000). The European Union Water Framework Directive—Directive 2000/60/EC of the European Parliament and of the Council Establishing a Framework for the Community Action in the Field of Water Policy.

[18] Loos R., Marinov D., Sanseverino I., Napierska D., Lettieri T. (2018). Review of the 1st Watch List under the Water Framework Directive and Recommendations for the 2nd Watch List.

[19] Baran W., Adamek E., Ziemiańska J., Sobczak A. (2011). Effects of the presence of sulfonamides in the environment and their influence on human health. J. Hazard. Mater..

[20] Felis E., Kalka J., Sochacki A., Kowalska K., Bajkacz S., Harnisz M., Korzeniewska E. (2020). Antimicrobial pharmaceuticals in the aquatic environment—Occurrence and environmental implications. Eur. J. Pharmacol..

[21] Liu L., Chen S., Xu K., Huang X., Liu C. (2021). Influence of hydraulic loading rate on antibiotics removal and antibiotic resistance expression in soil layer of constructed wetlands. Chemosphere.

[22] Miarov O., Tal A., Avisar D. (2020). A critical evaluation of comparative regulatory strategies for monitoring pharmaceuticals in recycled wastewater. J. Environ. Manag..

[23] Batt A.L., Furlong E.T., Mash H.E., Glassmeyer S.T., Kolpin D.W. (2017). The importance of quality control in validating concentrations of contaminants of emerging concern in source and treated drinking water samples. Sci. Total Environ..

[24] Khan H.K., Rehman M.Y.A., Malik R.N. (2020). Fate and toxicity of pharmaceuticals in water environment: An insight on their occurrence in South Asia. J. Environ. Manag..

[25] Bashir S., Peerzada O.H., Kaur N., Ali S. (2017). Reduction of pollution load in sewage water using aquatic macrophyte *Lemna minor* L. (duck weed). Environ. Ecol..

[26] Mitsou K., Koulianou A., Lambropoulou D., Pappas P., Albanis T., Lekka M. (2006). Growth rate effects, responses of antioxidant enzymes and metabolic fate of the herbicide Propanil in the aquatic plant *Lemna minor*. Chemosphere.

[27] Olmstead A.W., LeBlanc G.A. (2000). Effects of endocrine-active chemicals on the development of sex characteristics of *Daphnia magna*. Environ. Toxicol. Chem..

[28] Farré M., Barceló D. (2003). Toxicity testing of wastewater and sewage sludge by biosensors, bioassays and chemical analysis. Trends. Anal. Chem..

[29] Parvez S., Venkataraman Ch Mukherji S. (2006). A review on advantages of implementing luminescence inhibition test (*Vibrio fischeri*) for acute toxicity prediction of chemicals. Environ. Int..

[30] Sochacki A., Nowrotek M., Felis E., Kalka J., Ziembińska-Buczyńska A., Bajkacz S., Ciesielski S., Miksch K. (2018). The effect of loading frequency and plants on the degradation of sulfamethoxazole and diclofenac in vertical-flow constructed wetlands. Ecol. Eng..

[31] Nopens I., Capalozza C., Vanrolleghem P.A. (2001). Technical Report: Stability Analysis of a Synthetic Municipal Wastewater.

[32] Nowrotek M., Sochacki A., Felis E., Miksch K. (2016). Removal of diclofenac and sulfamethoxazole from synthetic municipal waste water in microcosm downflow constructed wetlands: Start-up results. Int. J. Phytoremediation.

[33] Góth L. (2001). A simple method for determination of serum catalase activity and revision of reference range. Clin. Chim. Acta.

[34] Misra H.P., Fridovich I. (1972). The role of superoxide anion in the autoxidation of epinephrine and a simple assay for superoxide dismutase. J. Biol. Chem..

[35] Bradford M.M. (1976). A rapid and sensitive method for the quantitation of microgram quantities of protein utilizing the principle of protein-dye binding. Anal. Biochem..

[36] (2007). Water Quality—Determination of the Inhibitory Effect of Waste Samples on the Light Emission of *Vibrio fischeri* (Luminescent bacteria Test)—Part 3: Method Using Freeze-Dried Bacteria.

[37] (2016). Water Quality—Marine Algal Growth Inhibition Test with *Skeletonema* sp. and *Phaeodactylum tricornutum*.

[38] Drzymała J., Kalka J. (2020). Elimination of the hormesis phenomenon by the use of synthetic sea water in a toxicity test towards *Aliivibrio fischeri*. Chemosphere.

[39] OECD (2004). Test No. 202: *Daphnia* sp. Acute Immobilisation Test. OECD Guidelines for the Testing of Chemicals.

[40] OECD (2006). Test No. 221: *Lemna* sp. Growth Inhibition Test. OECD Guidelines for the Testing of Chemicals.

[41] Persoone G., Marsalek B., Blinova I., Törökne A., Zarina D., Manusadzianas L., Nalecz-Jawecki G., Tofan L., Stepanova N., Tothova L. (2003). A practical and user-friendly toxicity classification system with Microbiotests for natural waters and wastewaters. Environ. Toxicol..

[42] Ra J.S., Lee B.C., Chang N.I., Kim S.D. (2008). Comparative whole effluent toxicity assessment of wastewater treatment plant effluents using *Daphnia magna*. Bull. Environ. Contam. Toxicol..

[43] Gizińska-Górna M., Czekała W., Jóźwiakowski K., Lewicki A., Dach J. (2016). The possibility of using plants from hybrid constructed wetland wastewater treatment plant for energy purposes. Ecol. Eng..

[44] Iwasaki Y., Kotani K., Kashiwada S., Masunaga S. (2015). Does the choice of NOEC or EC10 affect the hazardous concentration for 5% of the species?. Environ. Sci. Technol..

[45] van Vlaardingen P., Traas T.P., Wintersen A., Aldenberg T. (2004). ETX 2.0. A Program to Calculate Hazardous Concentrations and Fraction Affected, Based on Normally Distributed Toxicity Data.

[46] Saeed T., Sun G. (2013). A lab-scale study of constructed wetlands with sugarcane bagasse and sand media for the treatment of textile wastewater. Bioresour. Technol..

[47] Bulc T.G., Ojstršek A. (2008). The use of constructed wetland for dye-rich textile wastewater treatment. J. Hazard. Mater..

[48] Davies L.C., Carias C.C., Novais J.M., Martins-Dias S. (2005). Phytoremediation of textile effluents containing azo dye by using *Phragmites australis* in a vertical flow intermittent feeding constructed wetland. Ecol. Eng..

[49] Zhang D.Q., Tan S.K., Gersberg R.M., Zhu J., Sadreddini S., Li Y. (2012). Nutrient removal in tropical subsurface flow constructed wetlands under batch and continuous flow conditions. J. Environ. Manag..

[50] Ávila C., Matamoros V., Reyes-Contreras C., Piña B., Casado M., Mita L., Rivetti C., Barata C., García J., Bayona J.M. (2014). Attenuation of emerging organic contaminants in a hybrid constructed wetland system under different hydraulic loading rates and their associated toxicological effects in wastewater. Sci. Total Environ..

[51] Vymazal J. (2007). Removal of nutrients in various types of constructed wetlands. Sci. Total Environ..

[52] Carranza-Diaz O., Schultze-Nobre L., Moeder M., Nivala J., Kuschk P., Koeser H. (2014). Removal of selected organic micropollutants in planted and unplanted pilot-scale horizontal flow constructed wetlands under conditions of high organic load. Ecol. Eng..

[53] de la Paz A., Salinas N., Matamoros V. (2019). Unravelling the role of vegetation in the attenuation of contaminants of emerging concern from wetland systems: Preliminary results from column studies. Water Res..

[54] Miller E.L., Nason S.L., Karthikeyan K.G., Pedersen J.A. (2016). Root uptake of pharmaceutical and personal care product ingredients. Environ. Sci. Technol..

[55] Li Y., Zhu G., Ng W.J., Tan S.K. (2014). A review on removing pharmaceutical contaminants from wastewater by constructed wetlands: Design, performance and mechanism. Sci. Total Environ..

[56] Dordio A.V., Carvalho A.J.P. (2013). Organic xenobiotics removal in constructed wetlands, with emphasis on the importance of the support matrix. J. Hazard. Mater..

[57] Hu X., Xie H., Zhuang L., Zhang J., Hu Z., Liang S., Feng K. (2021). A review on the role of plant in pharmaceuticals and personal care products (PPCPs) removal in constructed wetlands. Sci. Total Environ..

[58] Shukla A., Parde D., Gupta V., Vijay R., Kumar R. (2021). A review on effective design processes of constructed wetlands. Int. J. Environ. Sci. Technol..

[59] Gorgoglione A., Torretta V. (2018). Sustainable management and successful application of constructed wetlands: A critical review. Sustainability.

[60] Fernandez R., Colás-Ruiz N.R., Bolívar-Anillo H.J., Anfuso G., Hampel M. (2021). Occurrence and Effects of antimicrobials drugs in aquatic ecosystems. Sustainability.

[61] Gonzalez-Gonzalez R.B., Flores-Contreras E.A., Parra-Saldívar R., Iqbal H.N.M. (2022). Bio-removal of emerging pollutants by advanced bioremediation techniques. Environ. Res..

[62] Sági G., Bezsenyi A., Kovács K., Klátyik S., Darvas B., Székács A., Wojnárovits L., Takács E. (2018). The impact of H2O2 and the role of mineralization in biodegradation or ecotoxicity assessment of advanced oxidation processes. Radiat. Phys. Chem..

[63] Wang Y., Li J., Lei Y., Li X., Nagarajan D., Lee D.-J., Chang J.-S. (2022). Analysis of pollutants removal efficiency, cellular composition, and bacterial community. Bioresour. Technol..

[64] Xiong Q., Liu Y.S., Hu L.X., Shi Z.Q., Cai W.W., He L.Y., Ying G.G. (2020). Cometabolism of sulfamethoxazole by a freshwater microalga *Chlorella pyrenoidosa*. Water Res..

[65] Lushchak V.I. (2011). Environmentally induced oxidative stress in aquatic animals. Aquat. Toxicol..

[66] Lushchak V.I. (2014). Free radicals, reactive oxygen species, oxidative stress and its classification. Chem. Biol. Interact..

[67] Ahmad R., Jaleel C.A., Salem M.A., Nabi G., Sharma S. (2010). Roles of enzymatic and nonenzymatic antioxidants in plants during abiotic stress. Crit. Rev. Biotechnol..

[68] Alscher R.G., Erturk N., Heath L.S. (2002). Role of superoxide dismutases (SODs) in controlling oxidative stress in plants. J. Exp. Bot..

[69] Zhang X.B., Liu P., Yang Y.S., Chen W.R. (2007). Phytoremediation of urban wastewater by model wetlands with ornamental hydrophytes. J. Environ. Sci..

[70] Yan Q., Feng G., Gao X., Sun Ch Guo J.-S., Zhu Z. (2016). Removal of pharmaceutically active compounds (PhACs) and toxicological response of *Cyperus alternifolius* exposed to PhACs in microcosm constructed wetlands. J. Hazard. Mater..

[71] Pradhan A., Sahu S.K., Dash A.K. (2013). Changes in pigment content (chlorophyll and carotenoid), enzyme activities (catalase and peroxidase), biomass and yield of rice plant (*Oriza sativa*.L) following irrigation of rice mill wastewater under pot culture conditions. Int. J. Sci. Eng. Res..

[72] Lyubenova L., Schröder P. (2011). Plants for waste water treatment—Effects of heavy metals on the detoxification system of *Typha latifolia*. Bioresour. Technol..

[73] Rizzo L., Meric S., Guida M., Kassinos D., Belgiorno V. (2009). Heterogenous photocatalytic degradation kinetics and detoxification of an urban wastewater treatment plant effluent contaminated with pharmaceuticals. Water Res..

[74] Majewsky M., Wagner D., Delay M., Bräse S., Yargeau V., Horn H. (2014). Antibacterial activity of sulfamethoxazole transformation products (TPs): General relevance for sulfonamide TPs modified at the para position. Chem. Res. Toxicol..

[75] Punzi M., Nilsson F., Anbalagan A., Svensson B.-M., Jönsson K., Mattiasson B., Jonstrup M. (2015). Combined anaerobic-ozonation process for treatment of textile wastewater: Removal of acute toxicity and mutagenicity. J. Hazard. Mater..

[76] Vymazal J. (2011). Plants used in constructed wetlands with horizontal subsurface flow: A review. Hydrobiologia.

[77] Drzymała J., Kalka J. (2020). Ecotoxic interactions between pharmaceuticals in mixtures: Diclofenac and sulfamethoxazole. Chemosphere.

[78] Pascual-Benito M., Nadal-Sala D., Tobella M., Ballesté E., García-Aljaro C., Sabaté S., Sabater F., Martí E., Gracia C.A., Blanch A.R. (2020). Modelling the seasonal impacts of a wastewater treatment plant on water quality in a Mediterranean stream using microbial indicators. J. Environ. Manag..

[79] Rice J., Wutich A., Westerhoff P. (2013). Assessment of de facto wastewater reuse across the U.S.: Trends between 1980 and 2008. Environ. Sci. Technol..

[80] Fortney L., Podein R., Hernke M., Rakel D. (2018). Detoxification. Integrative Medicine.

